# N-doped graphene layers encapsulated NiFe alloy nanoparticles derived from MOFs with superior electrochemical performance for oxygen evolution reaction

**DOI:** 10.1038/srep34004

**Published:** 2016-09-23

**Authors:** Yi Feng, Xin-Yao Yu, Ungyu Paik

**Affiliations:** 1 Department of Energy Engineering, Hanyang University, Seoul 133-791, Korea

## Abstract

Water splitting, an efficient approach for hydrogen production, is often hindered by unfavorable kinetics of oxygen evolution reaction (OER). In order to reduce the overpotential, noble metal oxides-based electrocatalysts like RuO_2_ and IrO_2_ are usually utilized. However, due to their scarcity, the development of cost-effective non-precious OER electrocatalysts with high efficiency and good stability is urgently required. Herein, we report a facile one-step annealing of metal-organic frameworks (MOFs) strategy to synthesize N-doped graphene layers encapsulated NiFe alloy nanoparticles (NiFe@C). Through tuning the nanoparticle size and calcination temperature, NiFe@C with an average size of around 16 nm obtained at 700 °C exhibits superior OER performance with an overpotential of only 281 mV at 10 mA cm^−2^ and high durability. The facile synthesis method and excellent electrochemical performance show great potential of NiFe@C in replacing the precious metal-based electrocatalysts in the OER.

Hydrogen production through electrochemical water splitting is a promising way to get clean and renewable energy compared with fossil fuels^1^,^2^. However, the efficiency of H_2_ evolution is subject to another key half-reaction in water splitting-oxygen evolution reaction (OER), due to its sluggish kinetics feature^3^,^4^,^5^. Highly efficient electrocatalysts are thus extremely needed to promote the OER rate. Nowadays, IrO_2_ and RuO_2_ are the state-of-the-art OER electrocatalysts with the highest efficiency^1^,^2^. However, their high cost and scarcity greatly hinder their large-scale application. Therefore, it is highly desirable to develop new OER electrocatalysts which are both highly efficient and cost-effective. During the past decades, nickel-based electrocatalysts including nickel oxides/hydroxides especially those with Fe doping, Ni-Fe layered double hydroxide and nickel phosphide are regarded as promising candidates due to their good electrocatalytic activities^6^,^7^,^8^,^9^. Recently, a few reports showed that elemental Ni and Ni-metal alloys are also efficient OER electrocatalyst^10^,^11^. For example, Chen *et al*. reported a three-dimensional catalyst by using N-doped graphene films as scaffolds and nickel nanoparticles as building blocks for catalyzing OER^10^. Very recently, nitrogen-doped graphene layer encapsulated transition metals and their alloys were also investigated as promising OER catalysts with lower overpotential and promoted long-term stability by Bao *et al*.^11^. The graphene shell not only prevents the corrosion of metal nanoparticles but also immensely promotes the electron transfer. Moreover, nitrogen doping can significantly improve the OER performance of graphene layer encapsulated metal nanoparticles. However, the complex and expensive CVD approach and multi-step synthesis processes greatly hinder the large-scale commercialization. Therefore, it will be of great interest to develop a more facile method to synthesize N-doped graphene layers encapsulated metal or metal alloy nanoparticles for efficient OER.

Prussian blue analogue (PBA), as a subclass of crystalline metal-organic frameworks (MOFs) with diverse compositions and morphologies has been extensively investigated as precursors to synthesize various functional materials^12^,^13^,^14^,^15^. Especially, PBA-derived metal oxides/hydroxides and metal sulfides are reported as promising electrode materials for electrochemical energy storage and conversion applications^13^,^14^. Most recently, our group demonstrated that PBA-derived metal phosphides are efficient electrocatalysts for both hydrogen and oxygen evolution reactions^16^,^17^. In addition, as PBAs are rich in N-containing organic ligands (CN- groups) and transition metal cations, they are believed to be promising precursors to prepare highly nitrogen doped carbon with metal or metal alloy nanoparticles.

Herein, we develop a facile strategy to synthesize N-doped graphene layers encapsulated NiFe alloy (NiFe@C) nanoparticles by one-step annealing of Ni_3_[Fe(CN)_6_]_2_ Prussian blue analogue (NiFe-PBA) for OER, as schematically shown in Fig. 1. Ni and Fe cations from the precursor will be reduced to form NiFe alloy nanoparticles, while CN- group linkers will form N-doped graphene layers outside the alloy nanoparticles during annealing process under high temperature and Ar atmosphere. Furthermore, through tuning the size of NiFe alloy nanoparticles and annealing temperature, the OER performances are also optimized. As expected, the NiFe@C electrocatalyst with size of about 16 nm synthesized at 700 °C exhibit excellent catalytic activity and stability for OER.

## Results

### Synthesis of NiFe-PBA precursors

To obtain small sized NiFe@C nanoparticles, gel-like NiFe-PBA precursor (G-NiFe-PBA) is synthesized via a co-precipitation method and the crystal structure is examined by X-ray diffraction (XRD). All the diffraction peaks could be assigned to Ni_3_[Fe(CN)_6_]_2_ (JCPDS no. 82-2283) without any impurity (Supplementary Fig. 1). The gel-like character is confirmed by scanning electron microscopy (SEM) and transmission electron microscopy (TEM) measurements (Supplementary Fig. 2a,b). The atomic ratio of Ni to Fe is determined to be 3:2 according to the energy dispersion X-ray (EDX) spectrum in Supplementary Fig. 2c. Different amount of trisodium citrate dehydrate (TSC) is further introduced into the reaction system to synthesize middle and big sized NiFe-PBA nanocubes for utilizing as precursors to synthesize bigger sized NiFe@C nanoparticles. Here, TSC is used to coordinate with metal ions to slow the nucleation and control the crystal growth^12^. When 180 mg of TSC are used, NiFe-PBA nanocubes with size of 40 nm (denoted as M-NiFe-PBA) is obtained, as confirmed by SEM and XRD (Supplementary Figs 1 and 3a). NiFe-PBA nanocubes with size of 100 nm (denoted as B-NiFe-PBA) are synthesized with addition of 300 mg of TSC (Supplementary Figs 1 and 3b). For comparison, Ni[Ni(CN)_4_] Prussian blue analogue (NiNi-PBA) precursor with nanoplate structure (Supplementary Figs 1 and 4) is also synthesized to obtain N-doped graphene layers encapsulated nickel nanoparticles (Ni@C).

### Synthesis of NiFe@C nanoparticles

**Figure 2:**
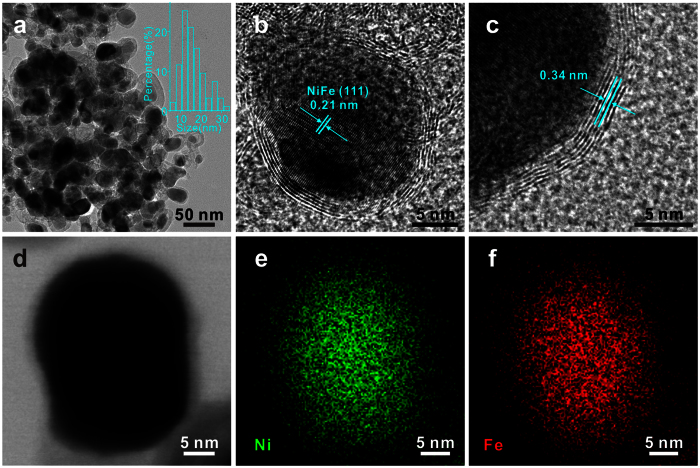
TEM characterizations of S-NiFe-700@C. TEM image (**a**) and HRTEM images (**b**,**c**) of S-NiFe-700@C. Inset of panel a: size distribution of S-NiFe-700@C. STEM (scanning transmission electron microscope) image (**d**) of S-NiFe-700@C and corresponding elemental mapping of Ni (**e**) and Fe (**f**).

**Figure 3:**
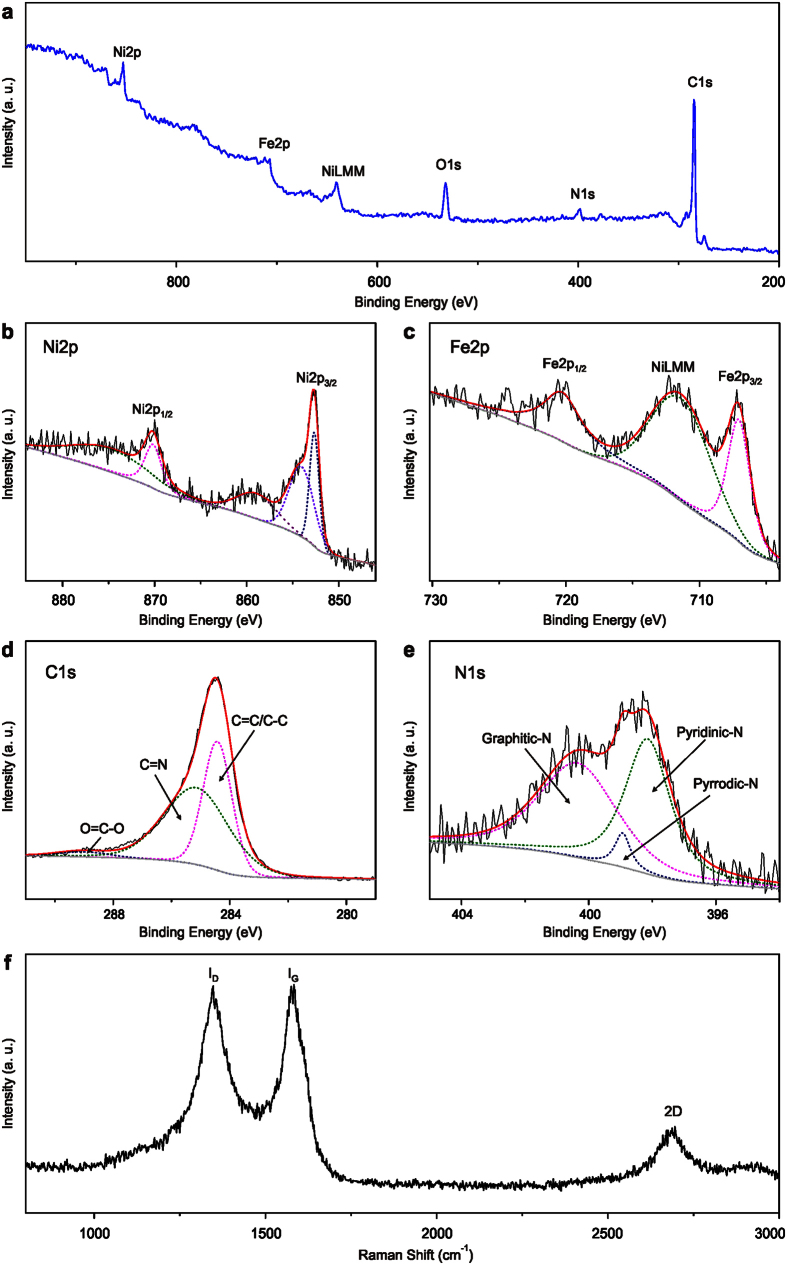
XPS spectrum of S-NiFe-700@C. XPS survey (**a**) and high resolution spectra of Ni2p (**b**) Fe2p (**c**) C1s (**d**) and N1s (**e**); Raman spectrum of S-NiFe-700@C (**f**).

G-NiFe-PBA is calcinated under 700 °C in Ar atmosphere to obtain small sized NiFe@C (denoted as S-NiFe-700@C). SEM and TEM images (Supplementary Fig. 5a and Fig. 2a) demonstrate the particle-like core-shell nanostructure with an average size of around 16 nm. The high-resolution TEM (HRTEM) image further demonstrates the core-shell structure and the lattice spacing of the core nanoparticles is determined to be 0.21 nm, corresponding to the (111) plane of NiFe alloy^11^ (Fig. 2b). The shell is determined to be graphene layers with an interlayer spacing of 0.34 nm (Fig. 2c) and the graphene shell is in the range of 6 to 28 layers (Fig. 2c and Supplementary Fig. 5b). Elemental mapping shows that Ni and Fe are uniformly distributed in S-NiFe-700@C nanoparticles, further confirming the alloy structure of NiFe (Fig. 2d–f). The molar ratio of Ni to Fe in S-NiFe-700@C is determined to be 1:1, as revealed by EDX spectrum (Supplementary Fig. 6). X-ray photoelectron spectroscopy (XPS) measurements are conducted to elucidate the chemical composition and nitrogen bonding configurations in S-NiFe-700@C. The survey XPS spectrum reveals the presence of Ni, Fe, N, C and O elements (Fig. 3a). The lower intensities of Ni and Fe compared to C indicate that the NiFe nanoparticles are almost fully confined to the interior of graphene shells. The metallic state of Ni in S-NiFe-700@C is confirmed by two principal peaks centered at 853 and 870 eV in high-resolution Ni2p XPS spectrum (Fig. 3b)^11^,^18^. The peaks located at 854 and 859 eV can be attributed to nickel oxides/hydroxides (owing to the exposure to air) and satellite, respectively^18^. The peaks at 707 and 720 eV in Fe2p XPS spectrum confirm the existence of metallic Fe (Fig. 3c) and peak at 711 eV comes from the Auger NiLMM^18^. The high-resolution C1s XPS spectrum could be deconvoluted into three peaks centered at 284.5, 285.1, and 289.0 eV, which could be indexed to C = C/C–C, C = N, and O = C–O, respectively (Fig. 3d)^15^. The N content in S-NiFe-700@C is 3.4% and the C content is 71.4%. In N1s XPS spectrum (Fig. 3e), the peaks located at 398, 399 and 400 eV can be assigned to pyridinic, pyrrolic and graphitic N, demonstrating the N-doping nature of the graphene layers^15^. The Raman spectrum of S-NiFe-700@C (Fig. 3f) displays the well-documented D, G and 2D bands located at 1347, 1583 and 2695 cm^−1^, respectively, which is the characteristic of graphene^11^. With the same calcination procedure, middle sized NiFe@C (33 nm, denoted as M-NiFe-700@C), big sized NiFe@C (61 nm, denoted as B-NiFe-700@C) and Ni@C can also be synthesized using M-NiFe-PBA, B-NiFe-PBA and NiNi-PBA as precursors, as confirmed by XRD, SEM, TEM, XPS and Raman measurements (Supplementary Figs 7–11). When G-NiFe-PBA is calcinated at 600 or 800 °C, NiFe@C with similar size to S-NiFe-700@C can also be obtained (Supplementary Figs 12–15). The NiFe@C synthesized at 600 and 800 °C is denoted as S-NiFe-600@C and S-NiFe-800@C, respectively.

### Electrocatalytic performance for OER

**Figure 4:**
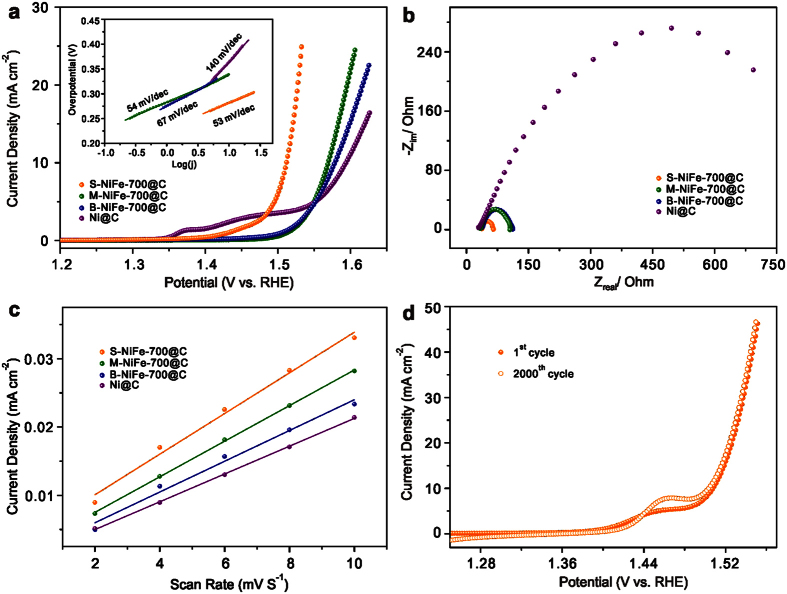
OER performance of NiFe@C and Ni@C nanoparticles synthesized at 700 °C. LSV curves (**a**) Tafel plots (inset of panel a), EIS Nyquist plots (**b**) and double layer capacitance (*C*_dl_) (**c**) of S-NiFe-700@C, M-NiFe-700@C, B-NiFe-700@C and Ni@C; Stability test of S-NiFe-700@C at a scan rate of 50 mV s^−1^ (**d**).

The electrocatalytic OER activity of the as-synthesized catalysts is investigated in alkaline solution (1 M KOH) in a standard three-electrode system. The linear sweep voltammetry (LSV) polarization curves show that all of the NiFe@C catalysts exhibit better OER performances than Ni@C, suggesting Fe has a significant effect on altering the electrocatalytic OER performance (Fig. 4a). In comparison with Ni@C, S-NiFe-700@C can remarkably reduce the overpotential from 364 to 281 mV for achieving a current density of 10 mA cm^−2^, while M-NiFe-700@C and B-NiFe-700@C need overpotential of 340 and 349 mV, respectively, to afford the same current density. The overpotential of S-NiFe-700@C is lower than that of IrO_2_/C at 310 mV and those of many other nickel-based catalysts and carbon encapsulated metal nanoparticles. A detailed comparison of different highly active electrocatalysts for OER is shown in Supplementary Table 1, further confirming the outstanding electrochemical performance of S-NiFe-700@C. In addition, the catalytic kinetics for oxygen evolution is investigated by Tafel plots (inset of Fig. 4a). The Tafel slope value of S-NiFe-700@C (53 mV dec^−1^) is lower than that of M-NiFe-700@C (54 mV dec^−1^), B-NiFe-700@C (67 mV dec^−1^), and Ni@C (140 mV dec^−1^) and comparable to those of the previously reported highly active OER electrocatalysts (Supplementary Table 1), revealing its favorable reaction kinetics. The electrode kinetics of these catalysts in the OER process is also investigated by electrochemical impedance spectroscopy (EIS) measurements at 1.55 V vs RHE. As can be seen from Fig. 4b, the charge-transfer resistance (*R*_ct_) values of these catalysts increase in the order S-NiFe-700@C < M-NiFe-700@C < B-NiFe-700@C < Ni@C, consistent with the OER performances. The electrochemically active surface area (ECSA) of these catalysts is estimated by measuring the electrochemical double-layer capacitance (*C*_dl_), as *C*_dl_ is proportional to the ECSA of electrocatalysts. The *C*_dl_ of S-NiFe-700@C is confirmed to be 2.98 mF cm^−2^, which is higher than that of M-NiFe-700@C (2.61 mF cm^−2^), B-NiFe-700@C (2.25 mF cm^−2^), and Ni@C (2.03 mF cm^−2^), by calculating the slope from the linear relationship of the current density against the scan rate (Supplementary Fig. 16 and Fig. 4c). The nitrogen content in Ni@C (4.2%) is higher than that in S-NiFe-700@C (3.4%), M-NiFe-700@C (1.3%) and B-NiFe-700@C (1.3%). On the other hand, in comparison with Ni@C, S-NiFe-700@C has similar size distribution, demonstrating the positive effect of Fe incorporation. The enhanced OER performance of S-NiFe-700@C compared to M-NiFe-700@C and B-NiFe-700@C can be attributed to the higher nitrogen content and the size effect. The smaller sized NiFe alloy nanoparticles in S-NiFe-700@C expose more catalytically active surface area for OER, contributing to its ultrahigh OER activity. In addition, the OER performance of S-NiFe-700@C is superior to that of graphene shells obtained by acid washing (Supplementary Fig. 17), demonstrating the critical role of NiFe alloy nanoparticles. The S-NiFe-700@C can also catalyze hydrogen evolution reaction in alkaline solution although the property is less impressive (Supplementary Fig. 18).

The OER performances of S-NiFe@C synthesized at different temperatures are also compared, as shown in Supplementary Fig. 19a. It is obvious that S-NiFe-700@C shows higher electrocatalytic activity than S-NiFe-600@C and S-NiFe-800@C. Also, the semicircular diameter in the EIS of S-NiFe-700@C is much smaller than that of S-NiFe-600@C and S-NiFe-800@C (Supplementary Fig. 19b). The influence of loading mass of electrocatalysts on the OER performance is also investigated (Supplementary Fig. 20). The result shows that S-NiFe-700@C exhibits optimal performance with a mass loading of 0.286 mg cm^−2^ on the glassy carbon electrode.

**Figure 5:**
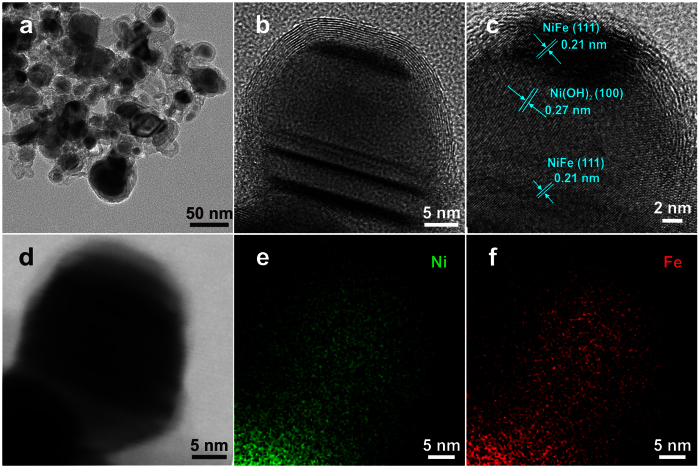
TEM characterizations of S-NiFe@700 after stability test. TEM image (**a**) and HRTEM images (**b**,**c**) of S-NiFe-700@C after stability test; STEM image (**d**) of S-NiFe700@C after stability test and corresponding elemental mapping of Ni (**e**) and Fe (**f**).

Stability is another important parameter to evaluate the performance of non-noble-metal based electrocatalysts in OER process. The durability of S-NiFe-700@C under continuous potential scanning conditions for 2000 cycles at a scan rate of 50 mV s^−1^ are shown in Fig. 4d. As observed, S-NiFe-700@C even shows better OER performance in the 2000^th^ cycle than the first cycle, suggesting their superior durability, which can be attributed to the activation of the electrocatalysts and the generation of more OER electrocatalytic active species such as nickel hydroxides (will be discussed in the following section). In addition, a slight peak emerges at around 1.46 V, which can be ascribed to the oxidation of Ni(II) to Ni(III). This oxidation may be attributed to the exposure of NiFe alloy nanoparticles during the long-term accelerated degradation test as some of the graphene layers may be destroyed during cycling. After the stability tests, we performed SEM, TEM, Raman, and XPS characterizations to check the morphology and structure changes of the S-NiFe-700@C catalysts (Fig. 5 and Supplementary Figs 21 and 22). Both the encapsulated NiFe alloy nanoparticles and most of the graphene layers are still well maintained after the OER durability test, as confirmed by TEM measurement (Supplementary Fig. 5a,b,d–f). HRTEM image reveals that Ni(OH)_2_ is generated on the surface of NiFe alloy nanoparticles (Fig. 5c). The high-resolution Ni2p XPS spectrum of S-NiFe-700@C (Supplementary Fig. 22a) after the stability test shows an intensity increase at around 855 eV, which can be assigned to either the Ni(OH)_2_ or NiOOH phase, and obvious intensity decreases at about 853 and 870 eV (corresponding to metallic Ni), demonstrating the surface oxidation of S-NiFe-700@C. The Auger peak of FKL at 861 eV comes from Nafion binder. Similar trend has been observed on the three peaks of the high-resolution Fe2p XPS spectrum located at 711, 707 and 719 eV, respectively (Supplementary Fig. 22b). In addition, a new peak located at ~723 eV corresponding to Fe oxides appears (Supplementary Fig. 22b). The above TEM and XPS results indicate that the surface of NiFe alloy nanoparticles is partially oxidized to nickel and iron oxides/hydroxides during the OER process. The high-resolution N1s XPS spectrum and Raman spectrum further demonstrate the N-doped graphene layers are well preserved (Supplementary Fig. 22c,d). Therefore, the eventually OER catalytically effective species is the NiFe/Ni and Fe oxides and hydroxides/N-doped graphene heterostructure. The OER catalytic mechanism of NiFe@C is similar to those of reported Ni_3_C/C nanoparticles^19^.

## Discussion

The outstanding OER electrocatalytic activities of the S-NiFe-700@C can be mainly attributed to the following aspects. (1) The nanoparticle morphology could provide a relatively large active surface area; (2) the Fe doped Ni core enables fast electron transport inside the electrocatalyst; (3) the encapsulated NiFe alloy nanoparticles could optimize the electronic structure of graphene layers^11^; (4) the *in situ* formed oxidized metal species on the surface of NiFe alloy nanoparticles may act as active sites for catalytic reactions; (5) The graphene shell on the surface of NiFe alloy nanoparticles would prevent the aggregation of nanoparticles and protect the core from corrosion. The synergistic effects from these aspects contribute to the enhanced OER performance of N-doped graphene layers encapsulated NiFe alloy nanoparticles.

In summary, we have developed a facile strategy to synthesize NiFe alloy nanoparticles encapsulated in N-doped graphene layers (NiFe@C). By controlling the nanoparticle size and annealing temperature, the OER performance can be optimized. Small sized NiFe@C nanoparticles (~16 nm) synthesized at 700 °C exhibit superior OER performance with lower overpotential, smaller Tafel slope and excellent durability. Most importantly, the MOF-derived facile one-step annealing approach may be extended to obtain various kinds of metal/metal alloy@C nanostructures.

## Methods

### Materials synthesis

The NiFe-PBA gels were synthesized by a simple precipitation method. In a typical procedure, 143 mg of NiCl_2_·xH_2_O were dissolved in 20 mL of deionized (DI) water to form solution A. At the same time, 132 mg of K_3_[Fe(CN)_6_] was dissolved in 20 mL of DI water to form solution B. Then, solutions A and B were mixed together under magnetic stirring for 1 min. The obtained mixed solution was aged for 12 h at room temperature. After collected by centrifugation and washed with water and ethanol, the precipitates were dried at 70 °C overnight. For the synthesis of NiFe-PBA nanocubes, 143 mg of NiCl_2_·xH_2_O and 180 or 330 mg of sodium citrate were dissolved in 20 mL of DI water to form solution C. Meanwhile, 132 mg of K_3_[Fe(CN)_6_] was dissolved in 20 mL of DI water to form solution D. Then, solutions C and D were mixed together under magnetic stirring for 1 min. The obtained mixed solution was aged for 12 h at room temperature. For the synthesis of NiNi-PBA nanoplates, 95 mg of NiCl_2_·xH_2_O was dissolved in 20 mL of deionized (DI) water to form solution E. 96 mg of potassium tetracyanidonickelate(II) was dissolved in 20 mL of DI water to form solution F. Then, solutions E and F were mixed under magnetic stirring for 1 min. The obtained mixed solution was aged for 12 h at room temperature. The as-prepared NiFe-PBA and NiNi-PBA were annealed at 600, 700, or 800 °C for 4 h with a heating temperature rate of 5 °C min^−1^ in argon atmosphere. N-doped graphene shells are synthesized by etching of S-NiFe-700@C in 1 M H_2_SO_4_ for 24 h.

### Materials characterizations

The crystal phase of the products was examined by XRD on a Rigaku D/MAX RINT-2000 X-Ray Diffractometer. Field-emission scanning electron microscope (FESEM; JEOL-7600F) and transmission electron microscope (TEM; JEOL, JEM-2100F) were used to examine the morphology of the samples. The composition of the samples and elemental mapping was analyzed by EDX attached to the TEM instrument. X-ray photoelectron spectrometer (XPS, VG microtech ESCA2000) was used for the analysis of the composition of the as-synthesized samples. Raman spectra were collected on a LabRam HR800 confocal micro-Raman system (JY Horiba).

### Electrochemical measurements

The OER activity was evaluated in a three-electrode configuration using a rotating disk electrode (RDE) (Autolab RDE/2, at a rotation speed of 1700 rpm) with an Autolab potentiostat/galvanostat (Model PGSTAT-72637) workstation at ambient temperature. A glassy carbon electrode (GCE) with a diameter of 3 mm was used as the support for the working electrode. The catalyst suspension was prepared by dispersing 5 mg of catalyst in 1 mL of solution containing 0.5 mL of DI water, 0.44 mL of ethanol and 60 μL of 0.5 wt.% Nafion solution followed by ultrasonication for 30 min. 4 μL of the catalyst suspension was pipetted onto the GCE surface using a micropipettor and then dried at ambient temperature. The catalyst loading amount is 0.286 mg cm^−2^ on the GCE. A Ag/AgCl (KCl saturated) electrode was used as the reference electrode and a platinum disc electrode was used as the counter electrode. Potentials were referenced to a reversible hydrogen electrode (RHE): E(RHE) = E(Ag/AgCl) + (0.2 + 0.059 pH)V. LSV was recorded in 1 M KOH (pH = 13.56) at a scan rate of 5 mV s^−1^ to obtain the polarization curves. The long-term stability tests were performed by continuous LSV scans with a sweep rate of 50 mV s^−1^. All the data presented were corrected for i*R* losses and background current. EIS was performed at overpotential of 320 mV with frequency from 0.1 to 100,000 Hz and an amplitude of 5 mV. The electrochemical double-layer capacitance was determined from the CV curves measured in a potential range of 1.10 V–1.15 V without redox processes according to the following equation: *C*_dl_ = *I*_c_/ν, where *C*_dl_, *I*_c_, and ν are the double-layer capacitance (F cm^−2^) of the electroactive materials, charging current (mA cm^−2^), and scan rate (mV s^−1^), respectively. Working electrodes were scanned for several potential cycles until the signals were stabilized, and then the CV data were collected. The plot of current density (at 1.12 V) against scan rate has a linear relationship and its slope is the double layer capacitance (*C*_dl_).

## Additional Information

**How to cite this article**: Feng, Y. *et al*. N-doped graphene layers encapsulated NiFe alloy nanoparticles derived from MOFs with superior electrochemical performance for oxygen evolution reaction. *Sci. Rep.* **6**, 34004; doi: 10.1038/srep34004 (2016).

## Supplementary Material

Supplementary Information

## Figures and Tables

**Figure 1:**
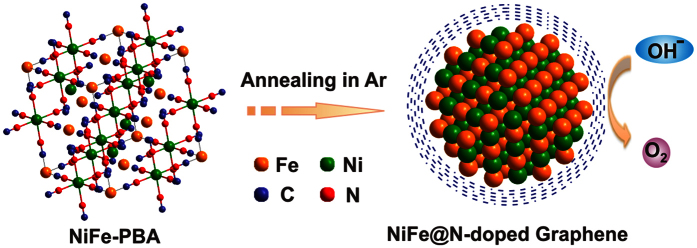
Synthetic route. Schematic synthesis route of N-doped graphene layers encapsulated NiFe alloy nanoparticles from NiFe-PBA for oxygen evolution reaction.
