# Supplementary material for: N-doped graphene layers encapsulated NiFe alloy nanoparticles derived from MOFs with superior electrochemical performance for oxygen evolution reaction
Source: Sci Rep. 2016 Sep 23;6:34004. doi: 10.1038/srep34004 (PMC5034270; doi:10.1038/srep34004)
Supplement: Supplementary Information [file srep34004-s1.pdf]

Supplementary Information

N-doped graphene layers encapsulated NiFe alloy nanoparticles derived from MOFs with superior electrochemical performance for oxygen evolution reaction

Yi Feng,[1] Xin-Yao Yu,[1]* Ungyu Paik[1]*

[1]Department of Energy Engineering, Hanyang University, Seoul 133-791, Korea.

*Corresponding author; Email: upaik@hanyang.ac.kr (U. P.) and yuxinyao@hanyang.ac.kr (X.-Y. Y.)

Supplementary Table 1. Comparison of OER performance of different non-noble metal-based electrocatalysts.

Electrode materials	Overpotential at 10 mA cm^{-2} (mV)	Tafel slope (mV dec^{-1})	References
**S-NiFe-700@C**	**281**	**53**	**This work**
Single layer graphene encapsulated NiFe alloy nanoparticles	280	70	[1]
NiFe/nanocarbon hybrids	330	45	[2]
Mesoporous NiFe alloy/CN_x	360	59	[3]
NiO_x nanoparticles	330	54	[4]
$Ni(OH)_2$ nanoparticles	300	53	[4]
α-$Ni(OH)_2$ hollow microspheres	331	42	[5]
NiFe-LDH nanosheets	302	40	[6]
NiFe-LDH/CNTs	247	31	[7]
Fe-doped nickel oxide nanocrystals	297	37	[8]
Ni-Co binary oxide nanoporous layers	325	39	[9]
Ni-Co oxide hierarchical nanosheets	340	51	[10]
Ni_2P nanoparticles	290	59	[11]
Ni_3N nanosheets	256	41	[12]
NiMo hollow nanorods array	310	47	[13]
Carbon layer coated Co nanoparticles	333	58	[14]
Co nanoparticles embedded porous N-rich carbon	370	76	[15]

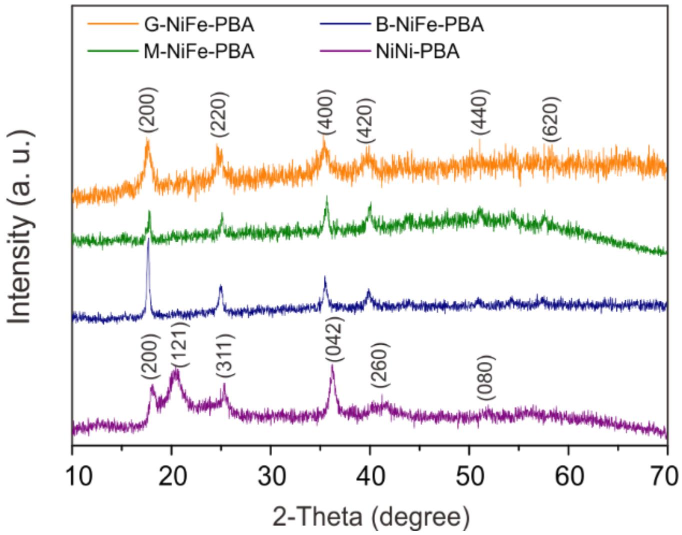

Supplementary Figure 1. XRD patterns of G-NiFe-PBA, M-NiFe-PBA, B-NiFe-PBA and NiNi-PBA.

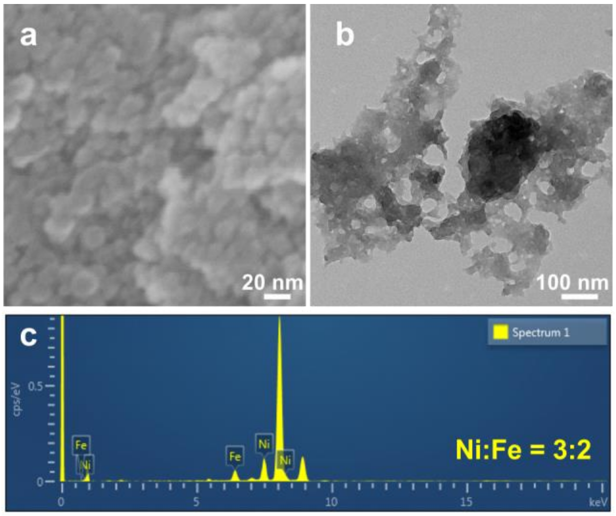

Supplementary Figure 2. SEM image (**a**), TEM image (**b**) and EDX spectrum (**c**) of G-NiFe-PBA.

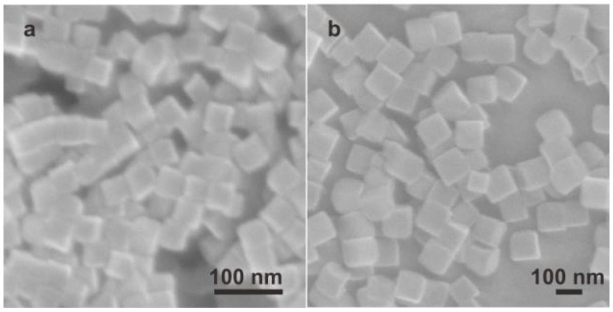

Supplementary Figure 3. SEM images of M-NiFe-PBA (**a**) and B-NiFe-PBA (**b**).

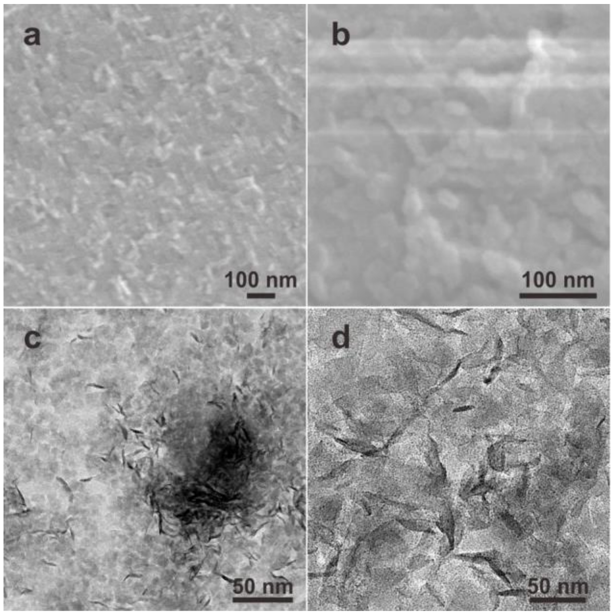

Supplementary Figure 4. SEM images (**a, b**) and TEM images (**c, d**) of NiNi-PBA.

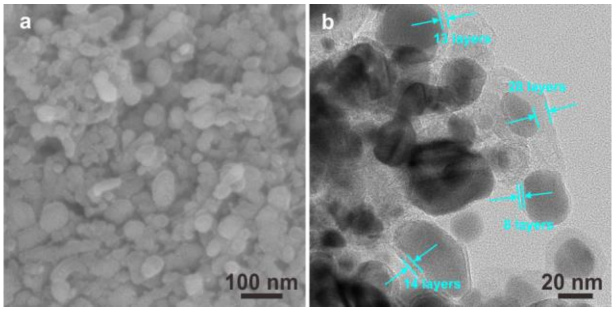

Supplementary Figure 5. SEM image (**a**) and TEM image (**b**) of S-NiFe-700@C.

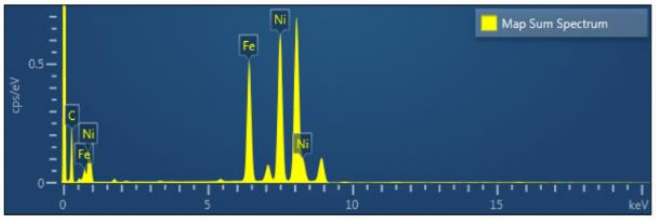

Element	Wt%	Atomic %
C	16.39	48.36
Fe	38.04	24.14
Ni	45.57	27.50
Total:	100.00	100.00

Supplementary Figure 6. EDX spectrum of S-NiFe-700@C.

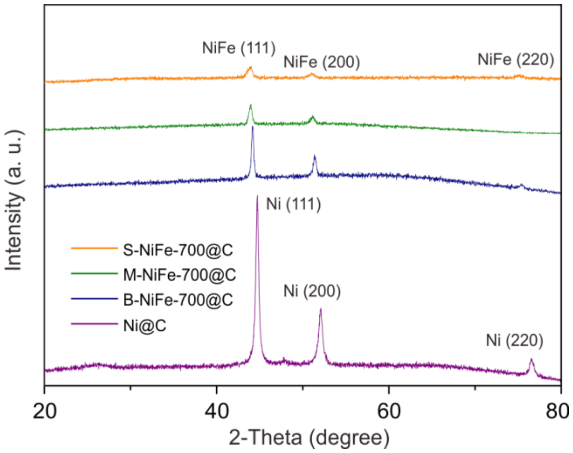

Supplementary Figure 7. XRD patterns of S-NiFe-700@C, M-NiFe-700@C, B-NiFe-700@C and Ni@C.

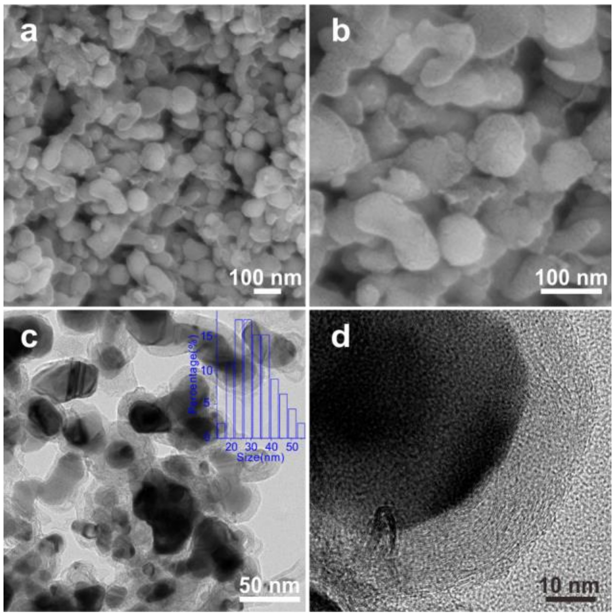

Supplementary Figure 8. SEM images (**a, b**) and TEM images (**c, d**) of M-NiFe-700@C. Inset of **c**: size distribution of M-NiFe-700@C.

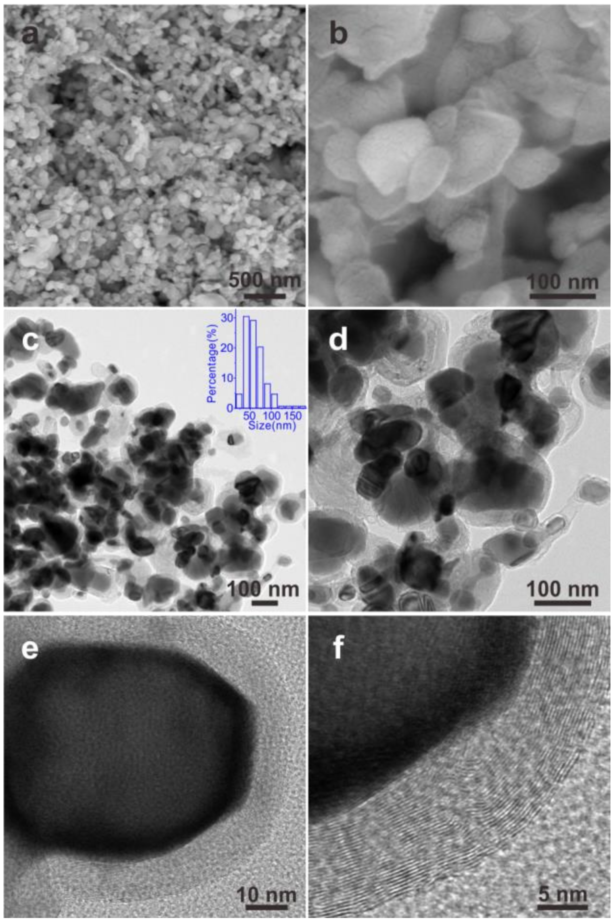

Supplementary Figure 9. SEM images (**a, b**) and TEM images (**c-f**) of B-NiFe-700@C. Inset of **c**: size distribution of B-NiFe-700@C.

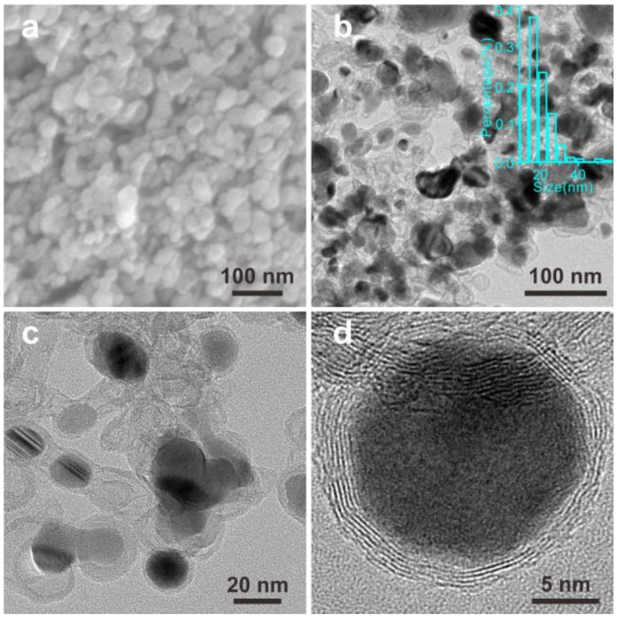

Supplementary Figure 10. SEM image (**a**) and TEM images (**b-d**) of Ni@C. Inset of **b**: size distribution of Ni@C.

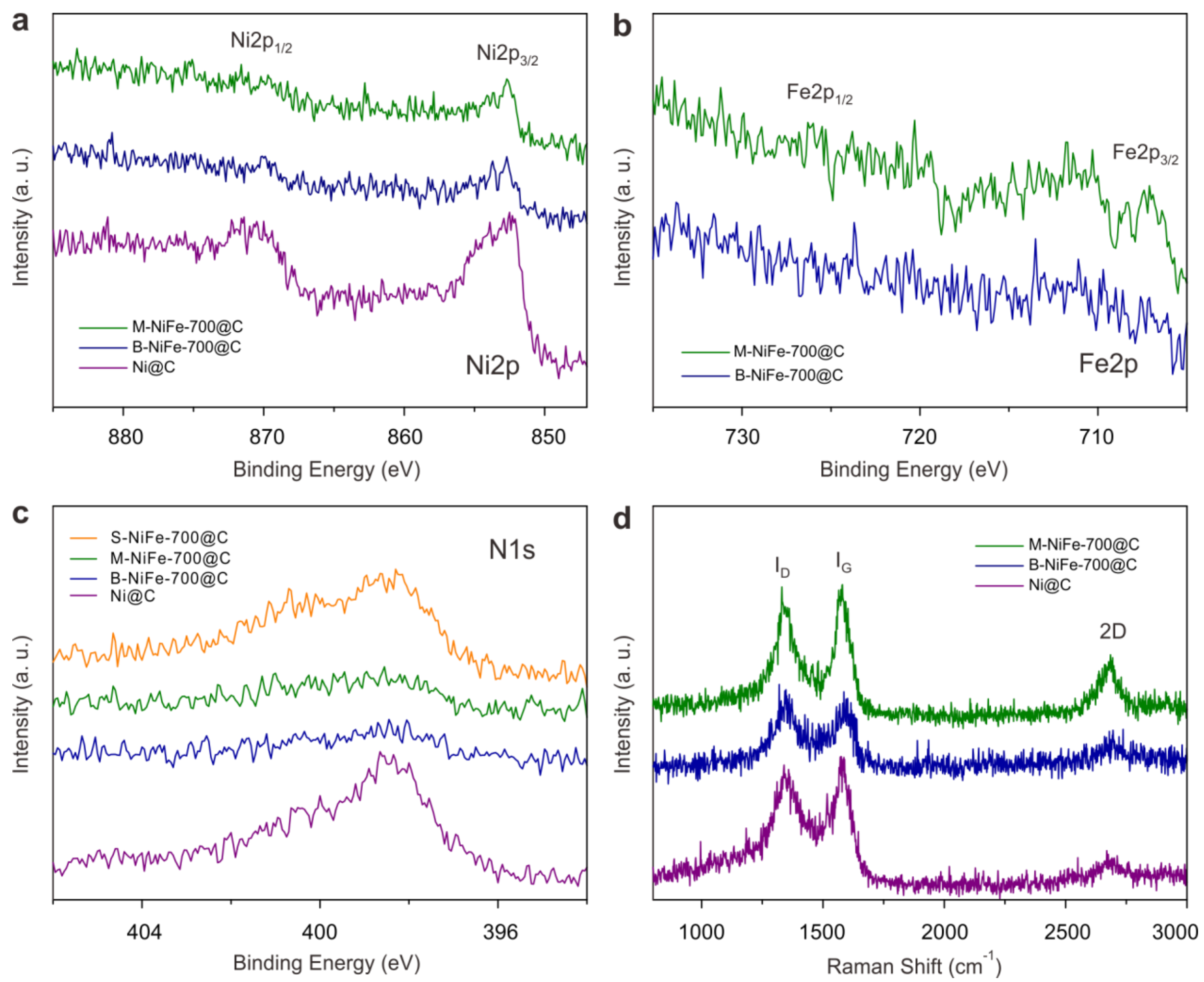

Supplementary Figure 11. Ni2p high resolution XPS spectra of M-NiFe-700@C, B-NiFe-700@C, and Ni@C (**a**); Fe2p high resolution XPS spectra of M-NiFe-700@C and B-NiFe-700@C (**b**); N1s high resolution XPS spectra of S-NiFe-700@C, M-NiFe-700@C, B-NiFe-700@C and Ni@C (**c**); Raman spectrum of M-NiFe-700@C, B-NiFe-700@C, and Ni@C (**d**).

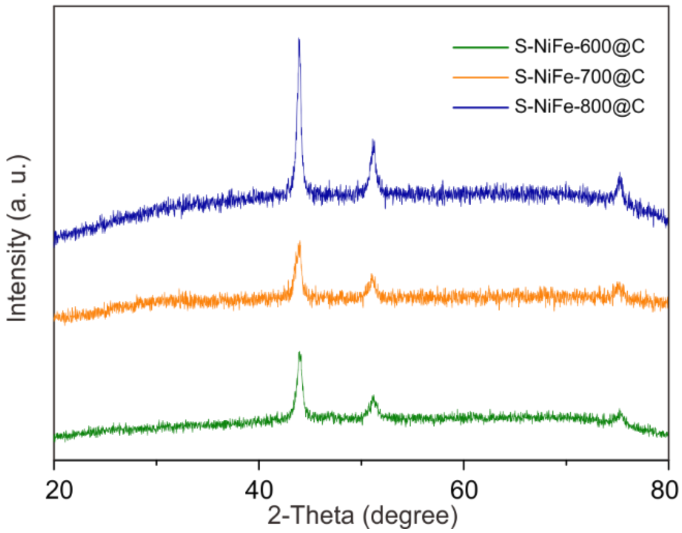

Supplementary Figure 12. XRD patterns of S-NiFe-600@C, S-NiFe-700@C and S-NiFe-800@C.

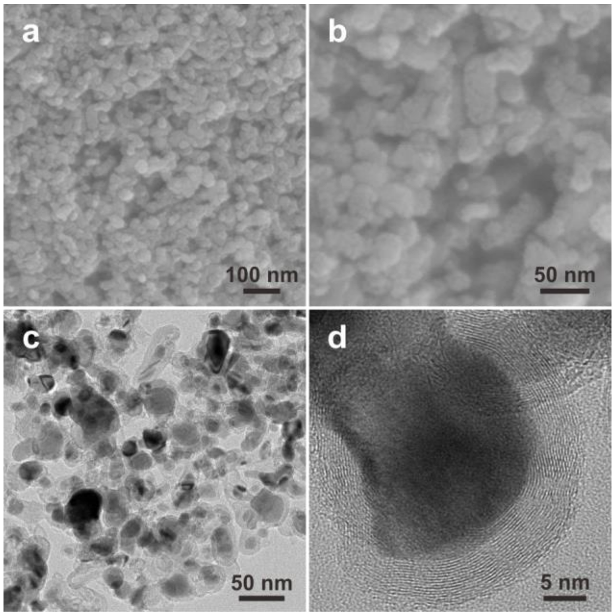

Supplementary Figure 13. SEM images (**a, b**) and TEM images (**c, d**) of S-NiFe-600@C.

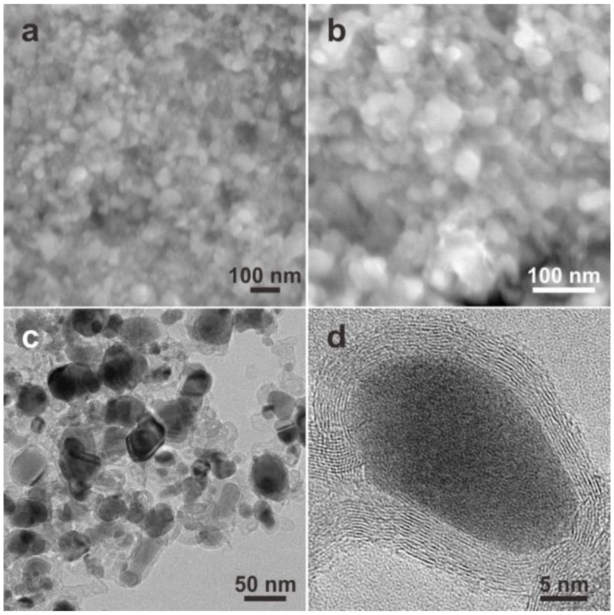

Supplementary Figure 14. SEM images (**a, b**) and TEM images (**c, d**) of S-NiFe-800@C.

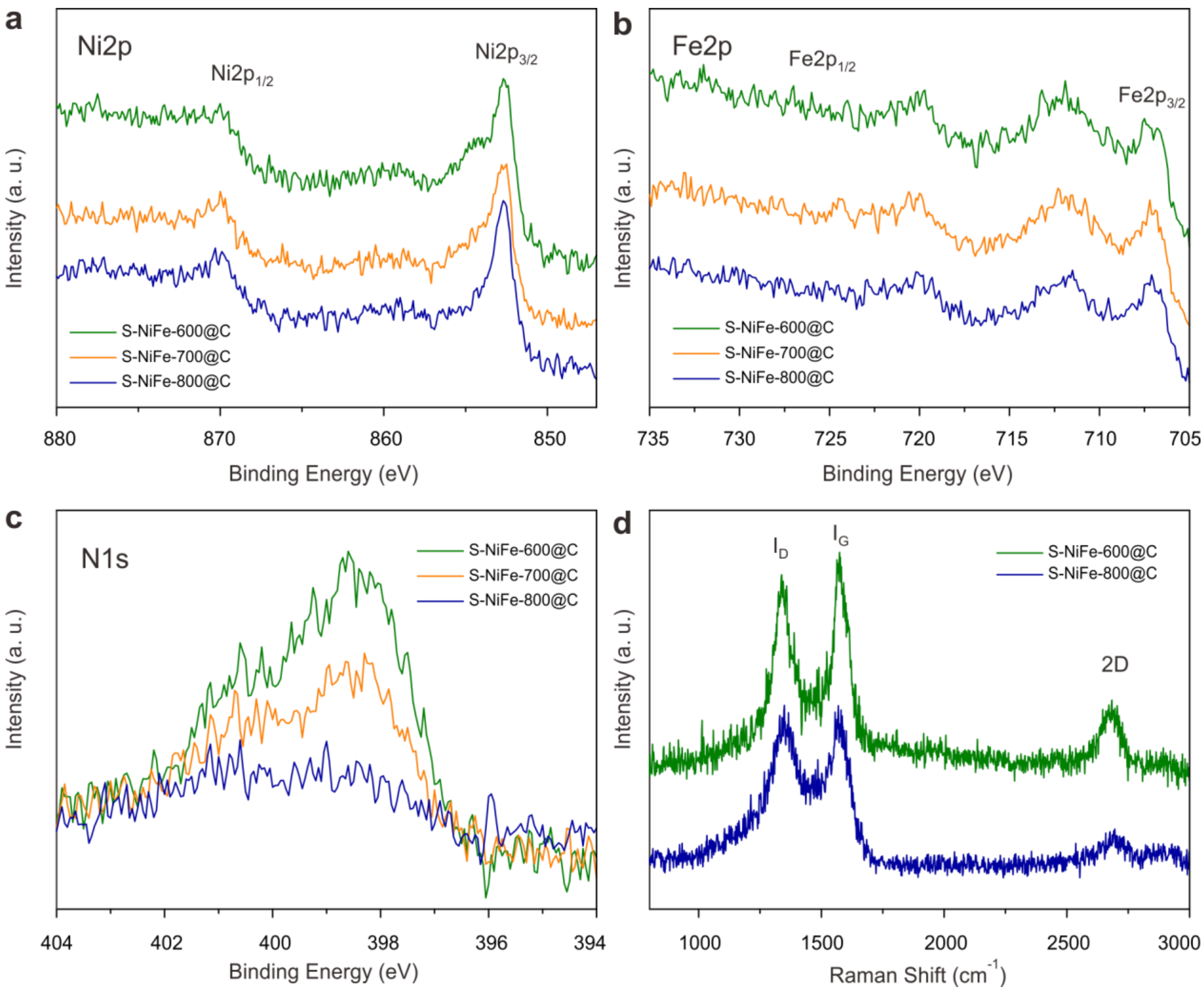

Supplementary Figure 15. Ni2p (**a**), Fe2p (**b**) and N1s (**c**) high resolution spectra of S-NiFe-600@C, S-NiFe-700@C and S-NiFe-800@C; Raman spectra of S-NiFe-600@C and S-NiFe-800@C (**d**).

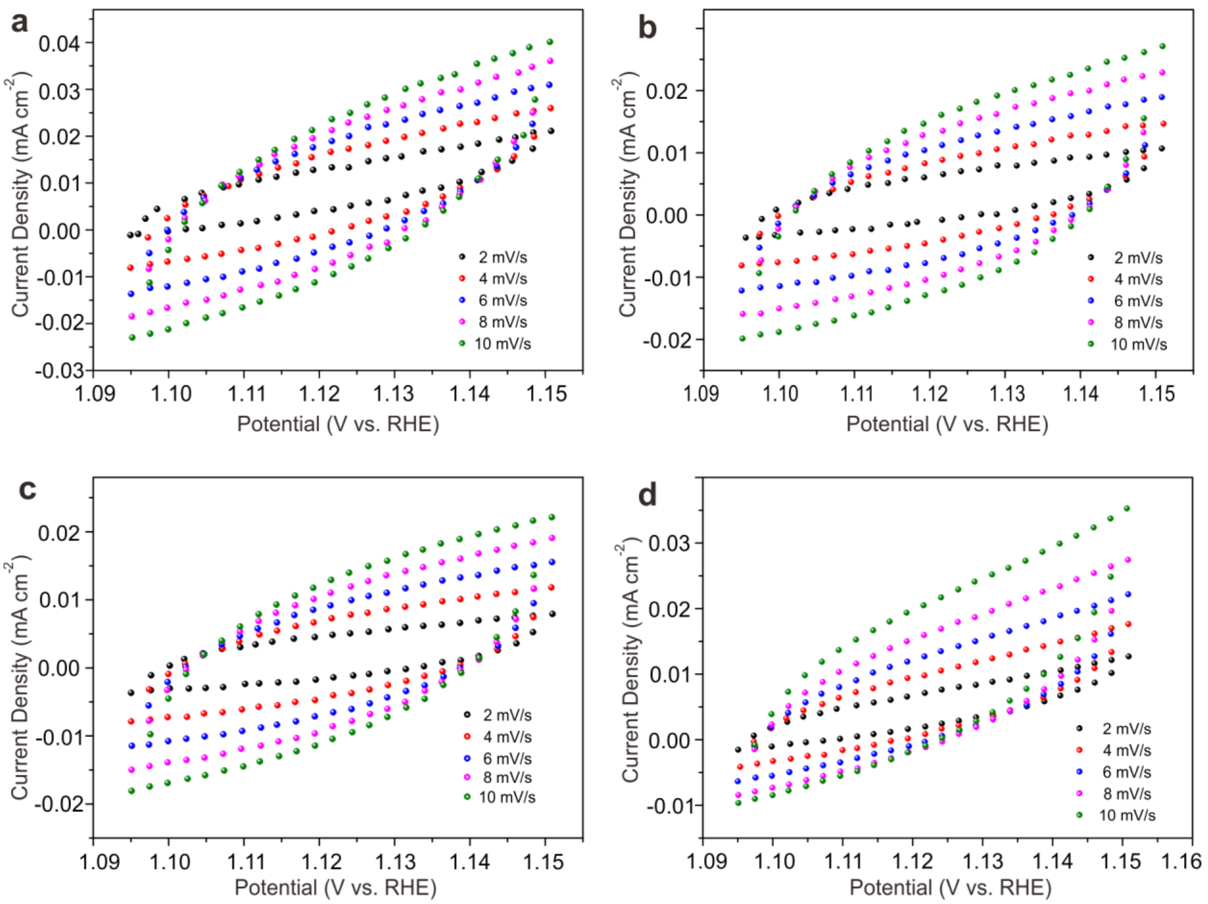

Supplementary Figure 16. CVs in the region of 1.10-1.15 V vs. RHE for S-NiFe-700@C (**a**), M-NiFe-700@C (**b**), B-NiFe-700@C (**c**) and Ni@C (**d**).

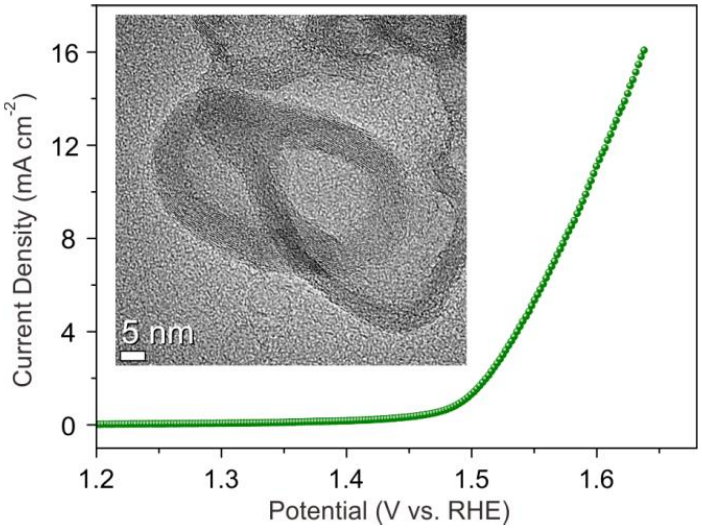

Supplementary Figure 17. LSV curve and TEM image (inset) of graphene shells obtained by acid washing of S-NiFe-700@C.

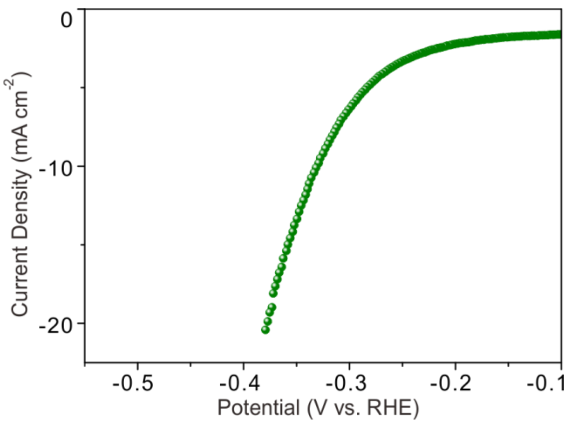

Supplementary Figure 18. LSV curve of S-NiFe-700@C for hydrogen evolution reaction in alkaline solution (1 M KOH).

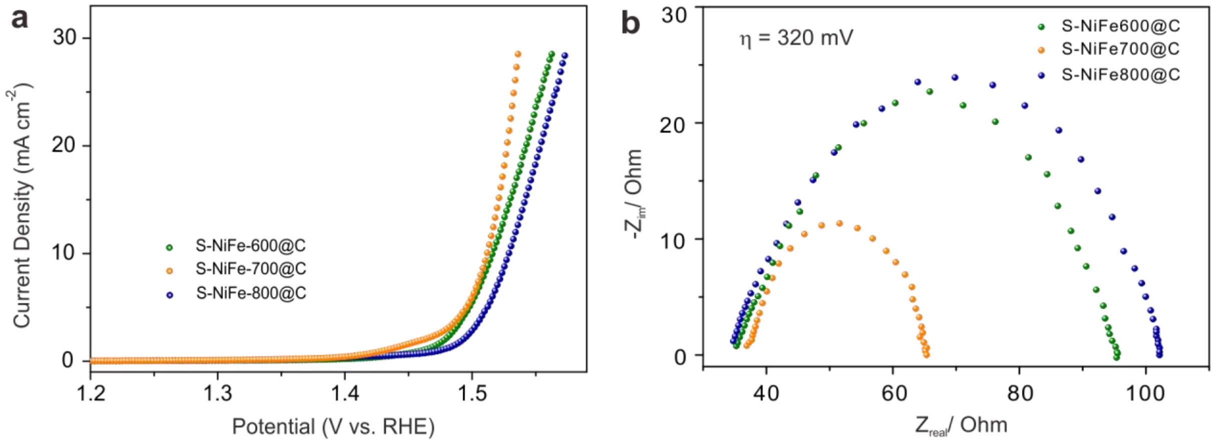

Supplementary Figure 19. LSV curves (**a**) and EIS Nyquist plots (**b**) of S-NiFe-600@C, S-NiFe-700@C and S-NiFe-800@C.

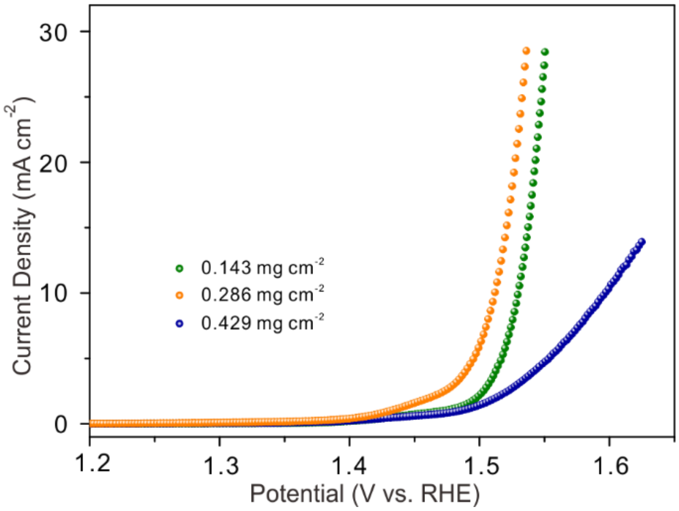

Supplementary Figure 20. LSV curves of S-NiFe-700@C with loadings of 0.143, 0.286, and 0.429 mg cm^{-2}.

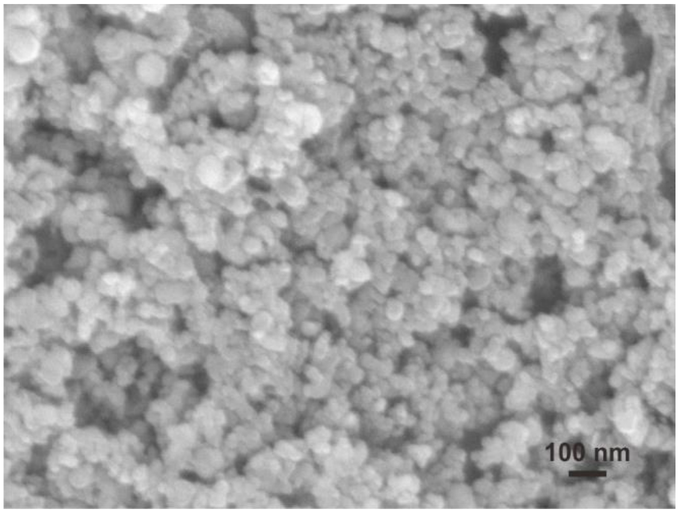

Supplementary Figure 21. SEM image of S-NiFe-700@C after stability test.

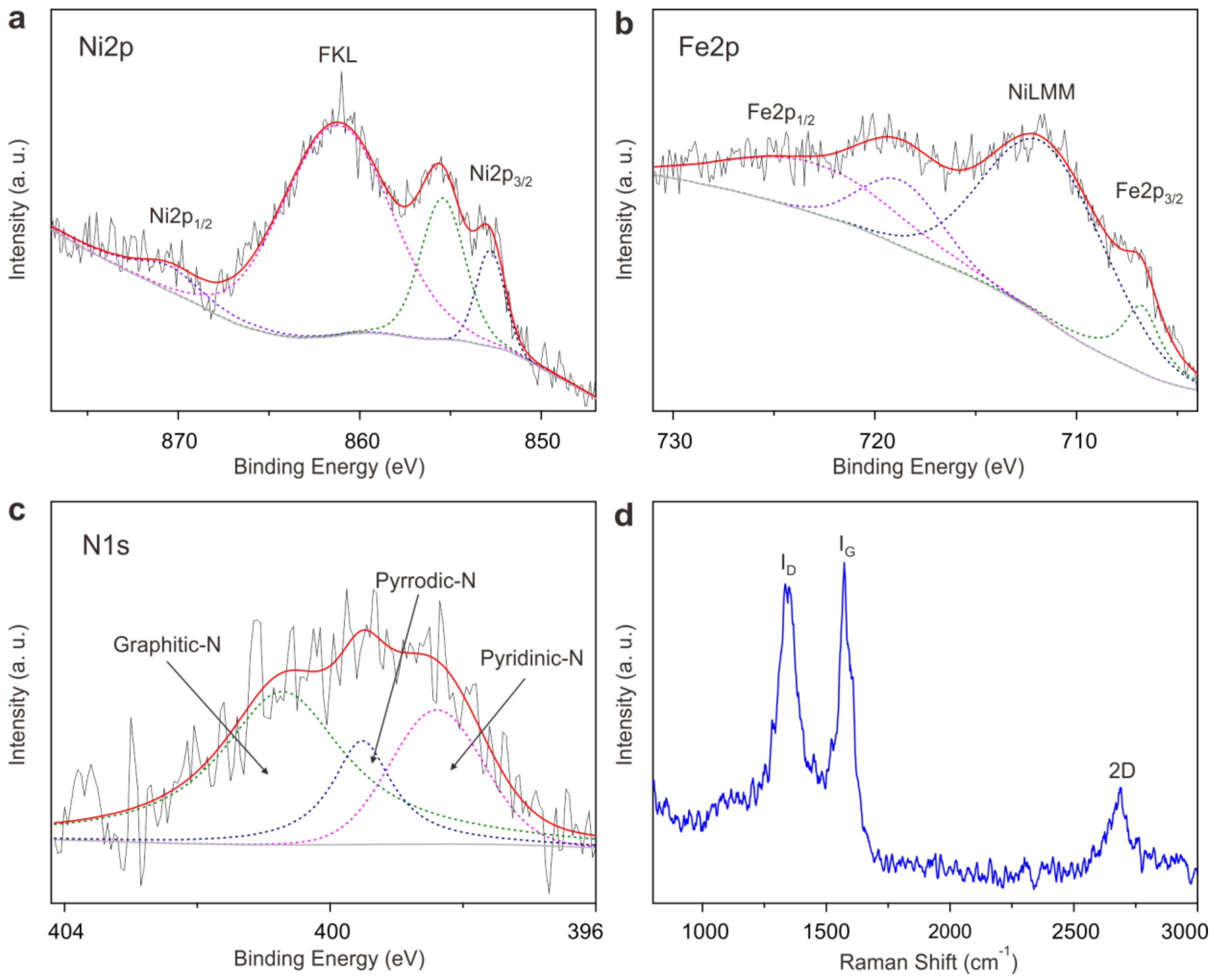

Supplementary Figure 22. Ni2p (**a**), Fe2p (**b**), N1s (**c**) high resolution XPS spectra and Raman spectrum (**d**) of S-NiFe-700@C after stability test.

References

1. Cui, X.J., Ren, P.J., Deng, D.H., Deng, J. & Bao, X.H. Single layer graphene encapsulating non-precious metals as high-performance electrocatalysts for water oxidation. *Energy Environ. Sci.* **9**, 123-129 (2016).
2. Zhang, X., *et al.* Facile Synthesis of Nickel-Iron/Nanocarbon Hybrids as Advanced Electrocatalysts for Efficient Water Splitting. *ACS Catal.* **6**, 580-588 (2016).
3. Ci, S.Q., *et al.* Rational design of mesoporous NiFe-alloy-based hybrids for oxygen conversion electrocatalysis. *J. Mater. Chem. A* **3**, 7986-7993 (2015).
4. Stern, L.A. & Hu, X.L. Enhanced oxygen evolution activity by NiOx and Ni(OH)(2) nanoparticles. *Faraday Discuss.* **176**, 363-379 (2014).
5. Gao, M.R., *et al.* Efficient Water Oxidation Using Nanostructured alpha-Nickel-Hydroxide as an Electrocatalyst. *J. Am. Chem. Soc.* **136**, 7077-7084 (2014).
6. Song, F. & Hu, X.L. Exfoliation of layered double hydroxides for enhanced oxygen evolution catalysis. *Nat. Commun.* **5**, 4477 (2014).
7. Gong, M., *et al.* An Advanced Ni-Fe Layered Double Hydroxide Electrocatalyst for

Water Oxidation. *J. Am. Chem. Soc.* **135**, 8452-8455 (2013).
8. Fominykh, K., *et al.* Iron-Doped Nickel Oxide Nanocrystals as Highly Efficient Electrocatalysts for Alkaline Water Splitting. *ACS Nano* **9**, 5180-5188 (2015).
9. Yang, Y., Fei, H.L., Ruan, G.D., Xiang, C.S. & Tour, J.M. Efficient Electrocatalytic Oxygen Evolution on Amorphous Nickel-Cobalt Binary Oxide Nanoporous Layers. *ACS Nano* **8**, 9518-9523 (2014).
10. Wang, H.Y., *et al.* Ni3+-Induced Formation of Active NiOOH on the Spinel Ni-Co Oxide Surface for Efficient Oxygen Evolution Reaction. *Adv. Energy Mater.* **5**, 1500091 (2015).
11. Stern, L.A., Feng, L.G., Song, F. & Hu, X.L. Ni2P as a Janus catalyst for water splitting: the oxygen evolution activity of Ni2P nanoparticles. *Energy Environ. Sci.* **8**, 2347-2351 (2015).
12. Xu, K., *et al.* Metallic Nickel Nitride Nanosheets Realizing Enhanced Electrochemical Water Oxidation. *J. Am. Chem. Soc.* **137**, 4119-4125 (2015).
13. Tian, J.Q., *et al.* Self-supported NiMo hollow nanorod array: an efficient 3D bifunctional catalytic electrode for overall water splitting. *J. Mater. Chem. A* **3**, 20056-20059 (2015).
14. Xiao, Q.Q., *et al.* A high-performance electrocatalyst for oxygen evolution reactions based on electrochemical post-treatment of ultrathin carbon layer coated cobalt nanoparticles. *Chem. Commun.* **50**, 13019-13022 (2014).
15. Li, X.Y., Niu, Z.G., Jiang, J. & Ai, L.H. Cobalt nanoparticles embedded in porous N-rich carbon as an efficient bifunctional electrocatalyst for water splitting. *J. Mater. Chem. A* **4**, 3204-3209 (2016).
